# Mechanism of resistance to mesotrione in an *Amaranthus tuberculatus* population from Nebraska, USA

**DOI:** 10.1371/journal.pone.0180095

**Published:** 2017-06-29

**Authors:** Shiv S. Kaundun, Sarah-Jane Hutchings, Richard P. Dale, Anushka Howell, James A. Morris, Vance C. Kramer, Vinod K. Shivrain, Eddie Mcindoe

**Affiliations:** 1 Syngenta Ltd., Jealott’s Hill International Research Centre, Bracknell, Berkshire, United Kingdom; 2 Syngenta, Research Triangle Park, NC, United States of America; 3 Syngenta, Vero Beach Research Center, Vero Beach, FL, United States of America; California State University Fresno, UNITED STATES

## Abstract

*Amaranthus tuberculatus* is a troublesome weed in corn and soybean production systems in Midwestern USA, due in part to its ability to evolve multiple resistance to key herbicides including 4-hydroxyphenylpyruvate dioxygenase (HPPD). Here we have investigated the mechanism of resistance to mesotrione, an important chemical for managing broadleaf weeds in corn, in a multiple herbicide resistant population (NEB) from Nebraska. NEB showed a 2.4-fold and 45-fold resistance increase to mesotrione compared to a standard sensitive population (SEN) in pre-emergence and post-emergence dose-response pot tests, respectively. Sequencing of the whole HPPD gene from 12 each of sensitive and resistant plants did not detect any target-site mutations that could be associated with post-emergence resistance to mesotrione in NEB. Resistance was not due to HPPD gene duplication or over-expression before or after herbicide treatment, as revealed by qPCR. Additionally, no difference in mesotrione uptake was detected between NEB and SEN. In contrast, higher levels of mesotrione metabolism via 4-hydroxylation of the dione ring were observed in NEB compared to the sensitive population. Overall, the NEB population was characterised by lower levels of parent mesotrione exported to other parts of the plant, either as a consequence of metabolism in the treated leaves and/or impaired translocation of the herbicide. This study demonstrates another case of non-target-site based resistance to an important class of herbicides in an *A*. *tuberculatus* population. The knowledge generated here will help design strategies for managing multiple herbicide resistance in this problematic weed species.

## Introduction

Hydroxyphenylpyruvate dioxygenase (HPPD, EC1.13.11.27) is a ubiquitous non-hemeoxygenase involved in the catabolism of the amino acid tyrosine [1, 2]. Additionally, it is a key enzyme in the synthesis of homogentisate, a precursor of plastoquinone and tocopherol in plants [3]. More specifically, HPPD catalyses the conversion of 4-hydroxyphenylpyruvate (HPP) into homogentisate in a complex reaction that involves decarboxylation of the 2-keto acid side chain of HPP followed by the hydroxylation of the aromatic ring and 1,2-rearrangement of the carboxymethyl group, the consumption of one oxygen molecule and the release of carbon dioxide [4, 5]. Tocopherol scavenges photosynthesis-derived reactive oxygen species, thereby preventing lipid peroxidation [6]. Recent work using deficient mutants have demonstrated that tocopherol also plays a role in other important physiological processes, such as germination, growth, and leaf senescence [7]. Plastoquinone is important as an electron acceptor for both phytoene desaturase in carotenoid biosynthesis and photosystem II [8]. Inhibition of HPPD results in the depletion of protective pigments leading to characteristic leaf bleaching and, ultimately, plant death [9].

Herbicides targeting HPPD represent one of the most recent and successful classes of inhibitors for the control of broadleaf and grass weeds in cereal crops [10]. They belong to an array of structurally diverse compounds that can be grouped into isoxazoles, pyrazolones and triketones [11]. Pyrazolinate was the first product to be marketed in the late 1970s and used for annual and perennial weed control in rice, although its true mode of action was not known at the time of launch [2]. The site of action of HPPD herbicides was determined much later, in the early 1990s, when the triketone inhibitor nitisinone was found to be a potent inhibitor of rat and mammalian HPPD [12, 13]. Subsequent biochemical and genetic studies demonstrated that structurally related triketone, isoxazole and pyrazolone compounds were competitive HPPD inhibitors in plants [14–17]. More recently, the precise mode of action of HPPD inhibitors was further resolved through co-crystallography studies revealing central roles played by two phenylalanine residues in the binding of benzoylpyrazole compounds to the target protein [5, 18].

Currently, HPPD herbicides have a market value of over 1.5 billion dollars with a major share for selective broadleaf and grass weed control in corn [19]. The four leading HPPD herbicides are mesotrione, isoxaflutole, tembotrione and topramezone, accounting for over 80% of sales. The demand for HPPD herbicides is projected to rise further, resulting from the development of HPPD tolerant soybean as an alternative for managing the increasing evolution of resistance to glyphosate in key weeds such as *Amaranthus* spp. [20, 21]. Several attributes have contributed to the commercial success of HPPD herbicides in corn agro-systems, including broad weed spectrum, flexibility in application timing, use in mixtures with compounds belonging to several major herbicide modes of action, and ability to synergise photosystem II herbicides [22–24].

As with other herbicides that have been used in large volumes over several years, however, resistance to HPPD inhibitors has evolved in two highly prolific and increasingly problematic *Amaranthus* species in Midwestern USA [25]. The first documented case of resistance was in an *Amaranthus tuberculatus* population from Illinois, called MCR, from a continuous seed corn production that had seen the repeated use of an HPPD herbicide for at least seven consecutive years [26]. Resistance to HPPD herbicides has been reported in three other *A*. *tuberculatus* populations from Iowa and Nebraska as well as one population each of *A*. *palmeri* from Kansas and Nebraska [25, 27–29]. In spite of its importance in corn agro-systems in the USA, to date the mechanism of resistance to an HPPD herbicide has been determined in only one *A*. *tuberculatus* population from Illinois [30]. The ability of this *A*. *tuberculatus* population to survive mesotrione was in part due to p450-mediated enhanced metabolism via 4-hydroxylation, mimicking one of the corn selectivity mechanisms [16]. Furthermore, metabolic resistance is reported to be supplemented by some level of HPPD gene over-expression in two *A*. *palmeri* populations from Kansas and Nebraska [31]. In this study, we have investigated resistance to four common HPPD herbicides used in US corn production systems in an *A*. *tuberculatus* population from Nebraska. We have also conducted detailed glasshouse and lab-based studies to determine the mechanism of resistance to mesotrione in this population.

## Materials and methods

### Plant material

The suspected resistant *A*. *tuberculatus* population (NEB) was sampled from a seed corn field in Nebraska (near Tarnov), USA in 2010. Seeds were collected from plants surviving a post-emergence field application rate of mesotrione (105 g ai ha^-1^). The owner gave permission to collect *A*. *tuberculatus* seeds from his land. The field received a soil application of s-metolachlor + atrazine before crop and weed emergence followed by a full labelled rate of tembotrione prior to the mesotrione rescue treatment. A standard sensitive biotype (SEN) was sourced from Herbiseed (Twyford, UK) and used for comparison in all glasshouse and laboratory-based studies.

### Confirmation of resistance to mesotrione

#### Seed treatment

To ensure maximum germination, the seeds from the SEN and NEB populations were sterilised in a 50:50 mix of sodium hypochlorite (Sigma) and water for 10 minutes. Then after, they were rinsed thoroughly with distilled water and placed on 0.4% plant agar (Duchefa) in 10 cm^2^ Petri- dishes (Fisher Scientific) and sealed with Parafilm (Sigma). The Petri-dishes were stored at 4°C for 21 days following which the seeds were dried on tissue paper overnight before sowing.

#### Pre- and post-emergence mesotrione dose response tests

The seeds of NEB and SEN were sown directly in sandy loam soil to a density that would provide around 15 plants per 10 cm-diameter pots. The pots were watered and maintained in a glasshouse providing a 16 H photoperiod of 180 μmol m^-2^ s^-1^ with temperatures of 24°C day and 18°C night and 65% relative humidity. For post-emergence application, the plants were treated with herbicides when they were 7 cm tall. For pre-emergence application, the seeds were sown as above and covered with soil prior to herbicide treatment. For both pre and post-herbicide treatments, mesotrione (Callisto^®^ 480 SC, Syngenta) was applied at 0.8, 1.6, 3.3, 6.7, 13.1, 26.3, 52.5, 105, 210, 420 and 840 g ai ha^-1^ using a track sprayer fitted with a Teejet nozzle delivering 200 L ha^-1^. All herbicide treatments included ammonium sulphate (Sigma) at 2.5% w/v and Agridex 1% v/v (Helena Chemical). Three replicate pots were used per herbicide treatment and population. Following herbicide treatment, the pots were arranged in a randomised block design and maintained in the same glasshouse conditions as before for 21 days, at which time they were assessed for percentage visual biomass reduction compared to an untreated control.

### Cross-resistance to other corn selective HPPD-inhibiting herbicides

To determine cross or multiple resistance to other HPPD herbicides, NEB and SEN plants were produced as described above and sprayed post-emergence with tembotrione (Laudis^®^, Bayer CropScience) at 50, 100, 200 and 400 g ai ha^-1^ and topramezone (Armezon^TM^, BASF) at 5, 10, 20 and 40 g ai ha^-1^, and pre-emergence with isoxaflutole (Balance^®^ Pro, Bayer CropScience) at 50, 100, 200 and 400 g ai ha^-1^. All herbicide treatments included Agridex at 1% v/v. Three replicate pots per herbicide treatment and population were included. Following herbicide treatment, the pots were arranged in a randomised block design and maintained in the same glasshouse conditions as before for 21 days, at which time they were assessed for percentage visual damage compared to an untreated control.

### Mechanism of resistance to mesotrione

Both potential target-site and non-target-site resistance mechanisms were investigated as part of this study. Target-site resistance studies consisted of (1) HPPD gene sequencing to identify potential mutations associated with resistance, (2) testing for HPPD gene duplication, (3) testing for constitutive and inducible HPPD gene over-expression following mesotrione treatment. Non-target-site resistance studies encompassed measuring relative uptake, translocation and metabolism of mesotrione between the sensitive and resistant populations.

#### HPPD gene sequencing

Twelve individual plants from the NEB population that survived the post-emergence application of mesotrione at 105 g ai ha^-1^ from the dose response test described above and twelve untreated plants of the SEN population were used in HPPD gene analysis. A RT-PCR approach was adopted to sequence the HPPD gene from the resistant NEB and sensitive SEN plants. For each of the 24 plants, 1 cm^2^ of leaf tissue was placed in a 24-well plate, frozen and then ground in a SPEX SamplePrep Geno/Grinder for 1 min at 1000 rpm following which 1.5 ml Tri-Reagent was added, mixed, incubated for 5 minutes and transferred to a 2 ml tube. The samples were centrifuged for 5 minutes at 10,000xg. Subsequently, RNA was isolated from the supernatant using the Zymo Research Direct-Zol RNA mini-prep kit.

First strand cDNA was generated using 200–1000 ng total RNA, 1ul oligo dT (12–18 nt) at 333 ng/μl, 4 μl 5X 1^st^ strand buffer, 2 μl 10 mM dNTPs, and 8 μl nuclease free water, heated to 65°C for 5 minutes and cooled on ice. Then, 2 μl 100 mM DTT and 1 μl Superscript II (200U/μl) (Life Technologies) were added and incubated at 42°C for 60 minutes in a thermocycler. Second strand DNA was produced using 1X OneTaq buffer, 200 μM dNTP, 1.2U OneTaq DNA polymerase, 50 ng each primer, 3 μl 1^st^ strand cDNA and nuclease free water to 50 μl. The forward (5’TCACTTTCTCTCTCATCATCTG3) and reverse primers (5’GCTGACAGCAATATTTTAGA3) were based on published upstream and downstream sequences of the 5’ and 3’ UTR of *HPPD* respectively in order to amplify the whole HPPD coding region (GenBank: JX259255). The PCR cycle conditions were: [94°C, 5 min], 35 x [94°C, 30 sec, 55°C, 30sec, 72°C, 1 min 40 sec], [72°C, 7 min]. A small aliquot was visualised on an agarose TBE gel stained with ethidium bromide to confirm successful amplification of a 1350 bp PCR product.

The PCR products were cleaned with ExoSAP-IT (Affymetrix) as described by the supplier. The reactions were submitted for full length sequencing using the following primers:

R10 GTGGTTTTGTGAAGATTTGAAGCA, IGB2466 ACCGTTTTCCAAGTGAATAG, IGB2467 TTTCCGTAGCTTACATACC, IGB2478 TACCAAATTCCTTACATCGCAC, IGB2515 GGGTTTCATGAGTTTGCTGAG, IGB2516 CTGAAAAGTTTCCCTTTCCA, IGB2523 TCACTTTCTCTCTCATCATCTG, IGB2524 GCTGACAGCAATATTTTAGA. The sequencing was completed using the vendor GeneWiz. Sequence data was assembled using the program Sequencher 4.7 (GeneCodes). The assembled DNA sequences were converted to protein sequences and aligned with the Vector NTI tool AlignX (Invitrogen).

#### HPPD gene duplication and constitutive or inducible gene over-expression

Seeds of NEB and SEN were sown in seed trays (modular soil) to a high density and covered with vermiculite. The seed trays were watered and maintained in a glasshouse providing a 16 h photo period of 180 μmol m^-2^ s^-1^ with temperatures of 24°C day and 18°C night and 65% relative humidity. One week later, 24 seedlings each from NEB and SEN were individually transplanted to 7 cm pots (to 50:50 soil) and maintained in the same glasshouse conditions as above. Each seedling was labelled individually for later identification. When the seedlings were 7 cm tall, 0.5 cm^2^ leaf tissue was sampled from each of NEB and SEN plants, placed in a Costar™ 96-Well Assay Block (Fisher Scientific) and frozen at -80°C for subsequent DNA analysis. A second lot of 0.5 cm^2^ leaf tissues from each of NEB and SEN plants were collected in 2 ml micro-centrifuge tubes and immediately frozen at -80°C for RNA extraction.

The 48 plants (24 each of NEB and SEN) were then treated with mesotrione (Callisto^®^ 480 SC) at 105 g ai ha^-1^ using a tracksprayer fitted with a Teejet nozzle delivering 200 L ha^-1^. The herbicide treatment included AMSat 2.5% w/v and Agridex 1% v/v. Forty-eight hours later, 0.5 cm^2^ leaf tissue was sampled from the 48 individual plants for future RNA analysis as described above. The plants were subsequently maintained in the same glasshouse conditions as before for 21 days at which time they were assessed for survivorship and visual biomass reduction compared to an untreated control. Twelve most sensitive SEN plants and 12 of the most resistant NEB individuals were chosen for HPPD gene copy number and overexpression experiments.

For gene duplication studies, DNA was extracted from leaf tissues from 12 each of the selected SEN and NEB plants collected prior to herbicide treatment using a Qiagen DNeasy Plant mini kit (Qiagen) as per the manufacturer’s instructions. DNA concentrations were determined using a NanoDrop ND-2000 spectrophotometer (Thermo Scientific) and then normalised to 20 ng/μl using autoclaved RO water.

For constitutive and inducible gene over-expression studies, RNA was extracted from the 12 SEN and 12 NEB selected plants using leaf tissues collected before and after mesotrione treatments respectively. RNA from individual plants was isolated using RNAzol^®^ RT (Sigma) as per the manufacturer’s instructions and treated with DNAse to remove DNA contamination. RNA concentrations were determined using a NanoDrop ND-2000 spectrophotometer (Thermo Scientific) and 550 ng total RNA was employed for each sample for cDNA synthesis using SuperScript® III Reverse Transcriptase First-Strand Synthesis System (ThermoFisher) with Oligo(dT) primers according to the manufacturer’s recommendations. A negative RT control was included for each RNA sample to ensure no amplification occurred through DNA contamination.

Gene copy number and expression of HPPD were determined relative to two control genes: acetolactate synthase (ALS) and carbamoyl phosphate synthetase (CPS). DNA from the SEN population was diluted in water 1:1, 1:2, 1:4, 1:8, 1:16 and 1:32 ratios and used to determine efficiency for each primer pair. The same dilutions were employed for determining the cDNA primer efficiency with SEN cDNA. The primer pairs were HPPD-forward 5’-TGGATCATGCTGTAGGGAATGTCCC-3’ with HPPD-reverse 5’-CATTCATTGGAAACAACACCATTTCATC-3’ and ALS-forward CGCTGCTCAAGGCTACGCTCG with ALS-reverse GCGGGACTGAGTCAAGAAGTGCATC. The CPS primers used were as described in Ma et al. [30]. Each pair of primers was prepared in a Power SYBR® green PCR master mix (Life Technologies Ltd) and used to amplify 1 μl of DNA and cDNA from each sample. A SEN-DNA bulk was made by mixing 10 μl DNA from the 12 individual SEN samples. Similarly SEN-cDNA before treatment and SEN-cDNA after treatment bulks were generated from corresponding mixtures of individual cDNAs. These three separate bulks were used as the reference controls in all qPCR runs.

The design for the qPCR experiment was as follows: (i) DNA and RNA samples from the 12 individual plants of each two populations were allocated to six 96-well qPCR plates in all; (ii) two biological replicates of each population were assayed per qPCR plate; (iii) each plant was tested in two technical replicates; and (iv) technical replicates from each plant sample were assigned to 18 wells on each plate (six wells for DNA, 6 for RNA before treatment and 6 for RNA following treatment). In these groups of six wells, two each were for HPPD, ALS and CPS gene analysis. The remaining wells were taken up by bulked control and negative control samples.

qPCR was conducted on a StepOnePlus™ Real-Time PCR System (Life Technologies Ltd.) with a program set to 95°C for 10 min followed by 40 cycles of 95°C for 15s followed by 60°C for 1 min, with a final step used to carry out the melt-curve analysis of 95°C for 15s then 60°C for 1 min followed by 0.3°C incremental increases every 15s until 95°C.

#### ^14^C mesotrione uptake and translocation

NEB and SEN plants were grown in individual 7 cm pots at 24/18°C day/night temperature, 65% relative humidity and irrigated as required. At the 4-leaf stage plants were treated with a [phenyl—U -^14^C]-mesotrione (0.6 MBq with specific activity 4.266 MBq/mg) solution supplemented with Agridex at an inclusion rate of 1% v/v. Unlabelled mesotrione was added to the radioactive solution to provide a treatment rate equivalent to 105 g ai ha^-1^ in a spray volume of 200 l ha^-1^. The mesotrione treatment was delivered in 20 x 0.2μl microdroplets (4μl total) applied in a 1 cm band across the middle of the adaxial surface of selected leaves to give 5,000 dps (1.2μg) per plant. The droplets were applied using a 10 μl Hamilton syringe with a 50 X multi-stepper mechanism. Four replicate plants were treated for each population and time points. The plants were sampled at zero time (5 minutes after the droplets had dried) for recovery comparisons and then at 6, 24, 48 and 72 hours after treatment. Individual plants were sectioned into treated area, meristem, rest of foliage, and stem and roots. Foliar surface residues were recovered by washing the treated leaf with 2 ml of acetonitrile 80:20 water containing 0.1% v/v Tween^®^ 20 (Sigma). Radioactivity in the leaf rinsate was quantified by liquid scintillation counting (LSC) using a Perkin Elmer Tricarb 2900TR. The different plant sections were then accurately quantified by sample oxidation using a Harvey OX 500 Biological Oxidiser with attached Zinnser robot (R. J. Harvey Instruments). The samples were subsequently quantified by LSC. Percentage uptake was determined by the total amount of radioactivity detected in the plants x 100/total radioactivity applied (washes at T0). Relative herbicide translocation was determined as: (sum of radioactivity from meristem + rest of foliage + stem and roots) x 100/total amount recovered from the plant (including the treated area).

#### Unlabelled mesotrione metabolism

Individual NEB and SEN plants were grown in 7 cm pots in the aforementioned glasshouse conditions. A treatment solution containing 525 μg/ml of active ingredient was prepared in deionised water using mesotrione (Callisto^®^ 480 SC) and Agridex at 1% v/v. At the 4-leaf stage, plants were treated with 20 x 0.2μl microdroplets of the solution (using a 10 μl Hamilton syringe with 50 x multi stepper mechanism) on the adaxial surface of the newest fully expanded leaf to provide 2.1 μg mesotrione per sample. Four replicate plants were used per time point and population. The plant tissues were sampled at 5 minutes (after the droplets had dried) for recovery comparisons and then at 6, 24, 48 and 72 hours after treatment. Foliar surface residues were recovered by washing the treated leaf with 2 ml of acetonitrile 80:20 water. A 1 ml aliquot was sampled from the washes for quantification by Liquid Chromatography-Mass Spectrometry (LC-MS).

The plant tissues were dissected into two parts, namely, treated leaf and rest of the plant. The treated leaves were placed in 2 ml MP Bio Fast prep tubes (containing garnet lysing matrix A and a ¼ ceramic sphere) with 1 ml acetonitrile 80:20 water and then frozen overnight. The rest of the plant was placed in 15 ml Fisher Brand centrifuge tubes with 3 ml of acetonitrile 80:20 water and frozen overnight. Foliar samples were removed from the freezer and allowed to thaw before being macerated. The treated leaf extracts were then centrifuged at 10,000 rpm for 15 minutes (using a Thermo Scientific PICO 17 centrifuge) and the rest of plants extracts were centrifuged at 3000 rpm for 10 minutes on a Heraeus 3L kit. A second 1 ml extraction was used for the treated leaf and the supernatants combined. Aliquots of 1 ml were removed from each supernatant and placed in 1.5 ml Chromacol crimp top LCMS vials for quantification of parent mesotrione and known metabolites: 4-hydroxymesotrione and AMBA [2-amino-4-(methylsulfonyl) benzoic acid] using an Acquity BEH C18 column attached to an Acquity Binary solvent manager and sample organiser with Thermo Scientific TSQ Vantage mass spectrometer. The samples were analysed using a reverse phase Acquity UPLC BEH C18 Column (130 Å, 1.7 μm, 2.1 mm X 50 mm). The mobile phase comprised two solutions: eluent A was 0.2% formic acid and eluent B was acetonitrile. The elution profile was as follows: step 1, A:B (95:5, v/v) isocratic for 0.5 min; step 2, A:B (95:5, v/v) to A:B (5:95, v/v) linear gradient for 4 min; step 3, A:B (5:95, v/v) isocratic for 0.4 min; step 4, A:B (5:95, v/v) to A:B (95:5, v/v) linear gradient for 0.1 min; and step 5, A:B (95:5, v/v) isocratic hold for 1 min to re-equilibrate the column. Parent mesotrione and known metabolites were quantified against matrix matched standard calibration curves.

Uptake was defined as the sum (mesotrione + detectable metabolites (4-OH mesotrione + AMBA)) x 100/total applied (washes at T0) in the whole plant. The relative amounts of mesotrione and its metabolites detected in the two plant sections were expressed as a percentage of total mesotrione absorbed.

### Statistical analysis

For the pre- and post-emergence dose-response tests on mesotrione, GR_50_ estimates were obtained for each population by fitting a least squares logistic regression model of the form:

$$P=\frac{100}{1+e^{-\beta (x-\mu )}}$$

where P denotes the visual percentage damage, x denotes log_10_(Rate), and μ and β denote the logGR_50_ and slope parameters respectively [32]. Resistance indices for NEB versus the SEN population were estimated as the ratio of the respective GR_50_ estimates and are quoted with 95% confidence limits. A statistically significant (P = 0.05) difference between the populations is concluded when the confidence interval for the resistance index does not include the value 1. This is equivalent to carrying out a t-test between the means of the logGR_50_ estimates of the populations in question. Analyses were carried out using SAS version 9.4.

For the qPCR data from the HPPD gene duplication and gene expression tests, separate statistical analyses were carried out on the different DNA and RNA measurements. Prior to analysis, the C_T_ values for each biotype and gene were averaged across the two technical replicates in each plate. When primer efficiencies are equal, testing for differences in HPPD gene copy number and HPPD gene expression between populations is equivalent to comparing populations in terms of the difference between the average C_T_ value for the HPPD gene and that of the ALS and CPS genes in turn. Consequently, the data were analysed using the analysis of variance model:

$$y_{ijk}=\mu +\gamma_{i}+\beta_{j}+\varepsilon_{k(ij)}$$

where y_ijk_ denotes the difference between the average C_T_ value for the HPPD gene and that of the ALS or CPS genes, for plant k of population j in plate i, μ denotes the overall true mean, γ_i_ denotes the effect of plate i, β_j_ denotes the effect of population j and ε_k(ij)_ denotes the random error associated with plant k of population j in plate i. The comparison between the populations is then equivalent to carrying out a t-test using the pooled plant-to-plant variation within plates and populations as the source of ‘error’ variation. The statistical significance of the population comparisons are summarised by p-values, a value of 0.05 or less indicating a statistically significant result.

The uptake, translocation and metabolism measurements were analysed by factorial analysis of variance using the model:

$$y_{ijk}=\mu +\beta_{i}+\gamma_{j}+\tau_{k}+{\left(\gamma \tau \right)}_{jk}+\varepsilon_{ijk}$$

where y_ijk_ denotes the transformed measured response for population j at time k in replicate i, μ is the overall true mean response, γ_j_ is the true effect of population j, τ_k_ is the true effect for time k, (γτ)_jk_ denotes the population-by-time interaction and ε_ijk_ is the error associated with each individual response. Since the data analysed were percentages, an arcsine transformation was applied prior to analysis in order to satisfy the assumption of variance homogeneity required for the validity of the pooled error estimate. Where the population-by-time interaction was not statistically significant, populations were compared averaged across time points. Otherwise, comparisons were made separately at each time point.

## Results

### Mesotrione resistance confirmation test

**Fig 1:**
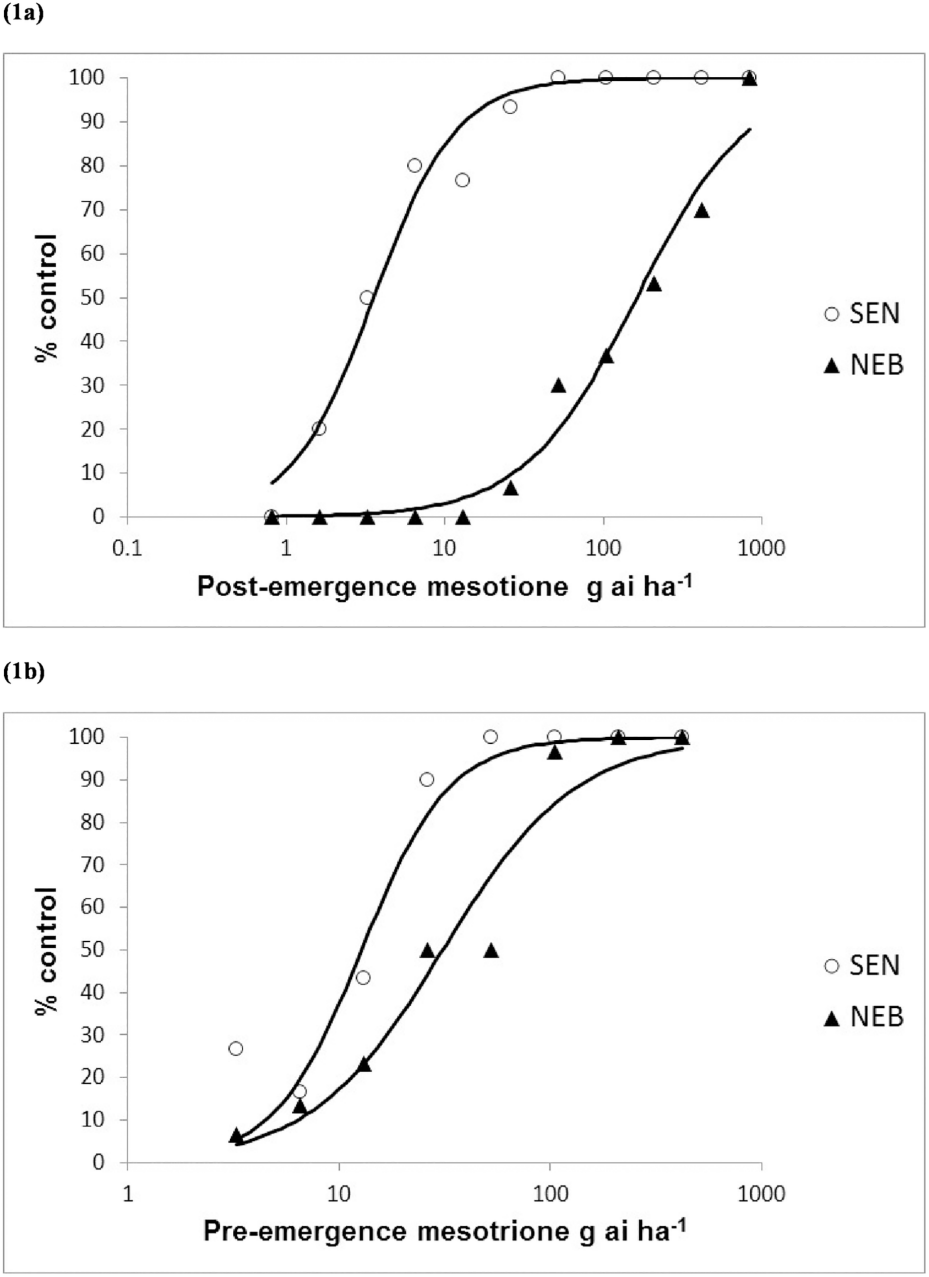
Mesotrione (1a) post-emergence and (1b) pre-emergence rate responses on the SEN and NEB *A*. *tuberculatus* populations.

The sensitive *A*. *tuberculatus* population SEN was fully controlled at 26 g ai ha^-1^ (0.25X field rate) mesotrione, demonstrating high efficacy of the herbicide when tested post-emergence under glasshouse conditions (Fig 1A). On the other hand, at this rate less than 10% biomass reduction was recorded for the resistant population NEB. At the commonly used post-emergence field rate of 105 g ai ha^-1^, mesotrione achieved only 37% control of NEB, thereby confirming field resistance to the HPPD herbicide in this population. Some clear but highly stunted survivors were still visible at 420 g ai ha^-1^ and full weed control was only attained at 840 g ai ha^-1^ mesotrione. The GR_50_ value for NEB was 162.1 (138.8–189.3) compared to 3.6 (3.1–4.1) for SEN amounting to a resistance index (RI) of 45.5 (37.1–55.8). When tested pre-emergence, lower levels of control were also observed for NEB as compared to SEN (Fig 1B). However, GR_50_ values between NEB and SEN were in the same order of magnitude and were estimated at 31.0 (25.1–38.2) and 12.8 (10.8–15.1) respectively. Consequently, the calculated resistance index was much lower at 2.4 (1.9–3.2), with full weed control of the NEB population achieved at around half the labelled field rate (105 g ai ha^-1^) of mesotrione applied pre-emergence.

### Cross or multiple resistance to other HPPD herbicides

**Fig 2:**
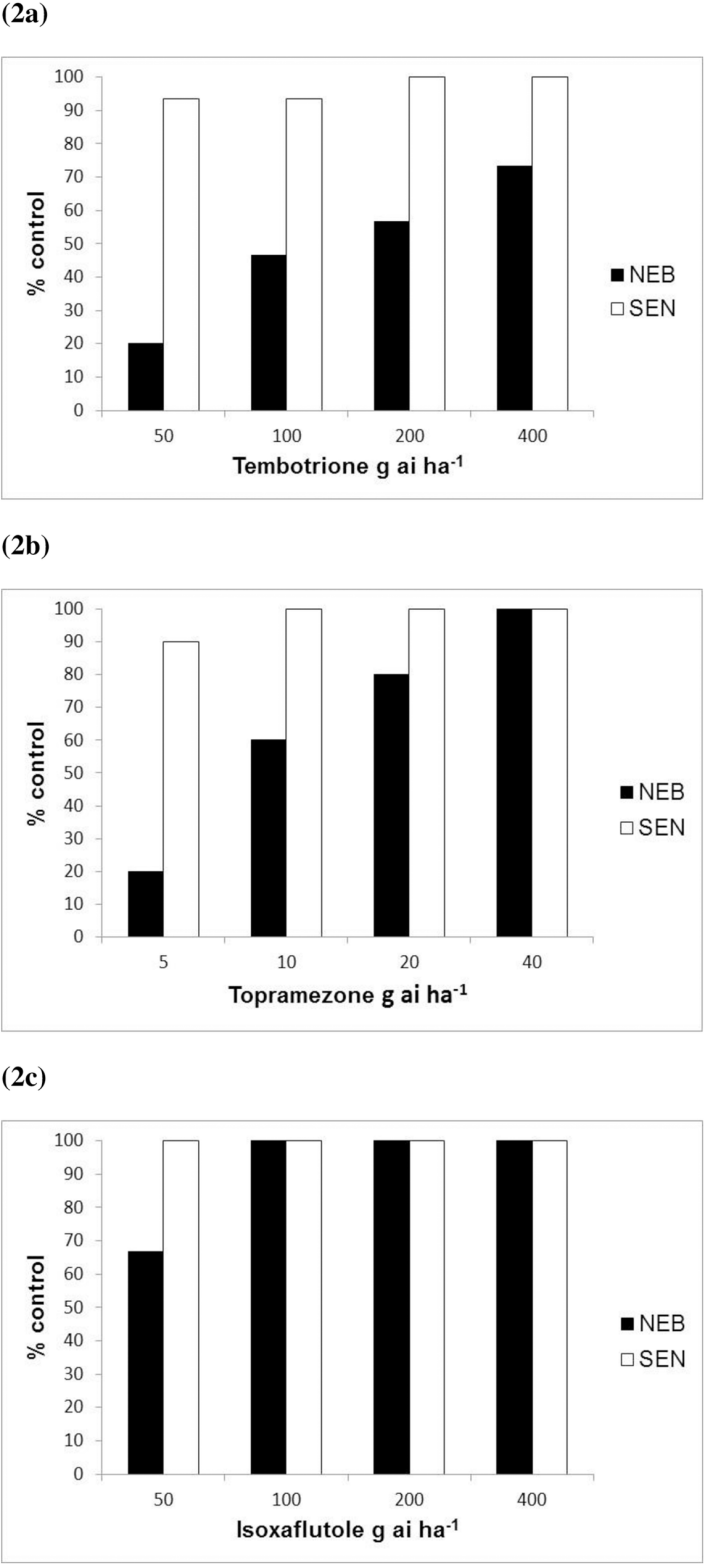
Cross-resistance profiles to three HPPD herbicides: (a) tembotrione (b) topramezone treated post-emergence and (c) isoxaflutole sprayed pre-emergence.

The cross or multiple resistance profile was determined for one each of a triketone, isoxazole and pyrazolone herbicide commonly used to control *Amaranthus* species pre-emergence (isoxaflutole) and post-emergence (tembotrione and topramezone) in corn agro-systems in the USA. All three herbicides were efficacious, achieving full control of the SEN population at half the recommended field rates and above (Fig 2A, 2B and 2C). Unsatisfactory control was observed for NEB with tembotrione and topramezone applied post-emergence, both providing only 20% biomass reduction at the rates that killed the sensitive population. In all cases clear survivors were identified at the recommended field rates for these two latter herbicides. Similarly, isoxaflutole applied pre-emergence provided lower levels of control on NEB compared to SEN at the discriminating rate of 50 g ai ha^-1^. The shift in the herbicide response was less pronounced compared to the two other herbicides tested post-emergence, with 100% control of NEB attained within the range of recommended field rates for this isoxazole herbicide.

### Mechanism of resistance to mesotrione

#### HPPD gene sequencing

Using primer pairs located on the 5’ and 3’ UTR of the HPPD gene, RT-PCR generated a 1350 bp fragment for all the 24 plants analysed. The 1305 translated region showed on average 99% and 80% identity to published *HPPD* sequences from *A*. *tuberculatus* (GenBank: JX259255) and *Beta vulgaris* (GenBank: XM_010690603) samples respectively, thereby supporting the identity of the target gene amplified in this study. Sequence comparison between the 12 SEN and 12 NEB individuals identified 52 nucleotide changes at 44 codon positions. One each of the SEN (accession number: KY689231) and NEB (accession number: KY689232) *HPPD* coding sequences were submitted to GenBank. Fifteen nucleotide changes were non-synonymous, resulting in amino acid mutations at 11 codon positions: Lys5Ile/Arg, Val25Ala, Thr33Asn/Lys, Lys35Glu, Arg81Ser/His, Cys149Phe, Glu196Asp, Arg200Gln, Asp242Glu, Met395Leu, Glu424Lys. Importantly, none of these mutations were consistently found in the resistant versus the sensitive plants, implying that a target gene mutation is not associated with resistance in the NEB population.

#### HPPD gene duplication and over-expression

**Fig 3:**
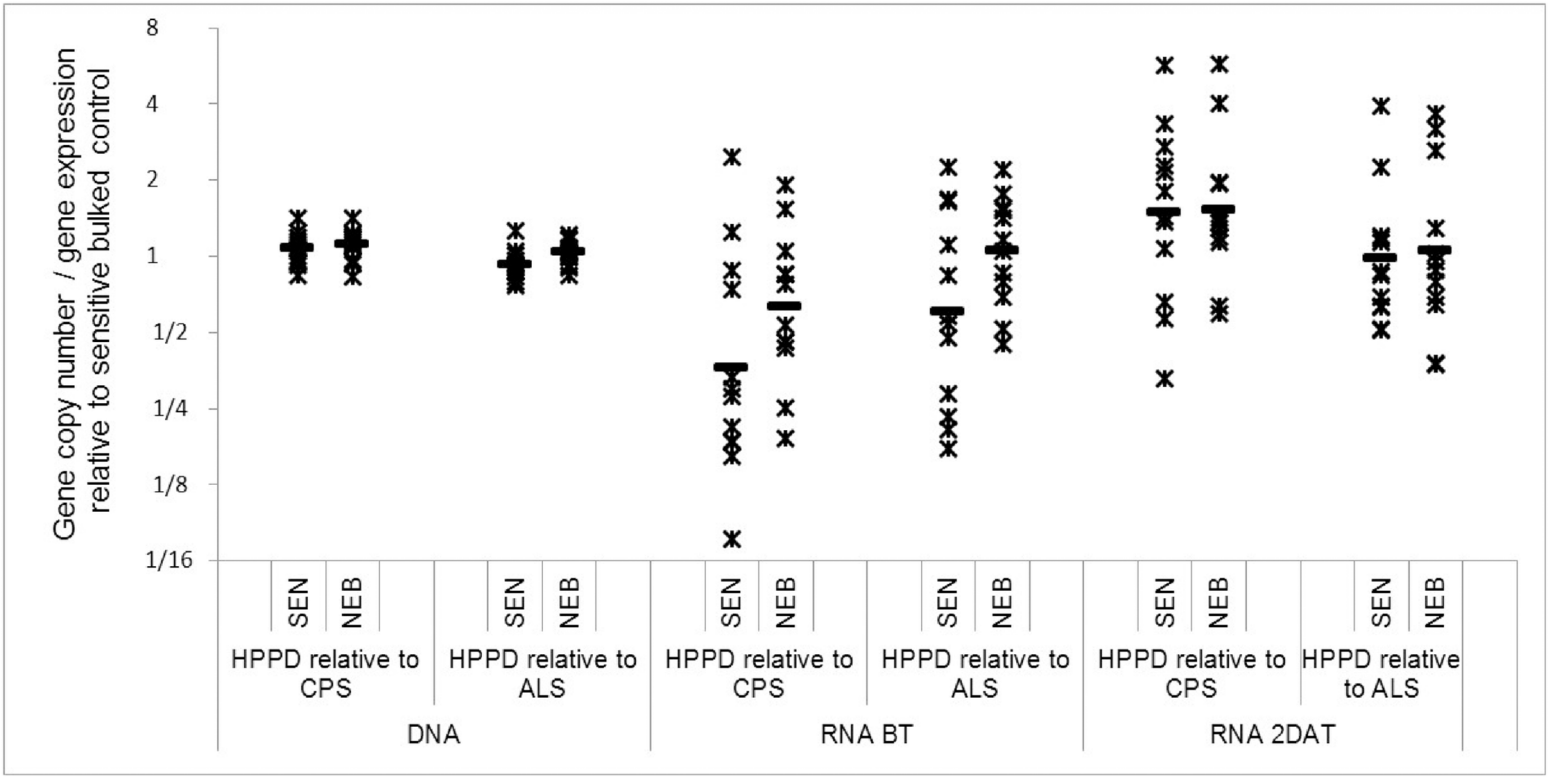
Scatter plot of the HPPD gene copy number and expression levels per SEN and NEB plant analysed relative to bulked control samples (BT: before treatment; 2 DAT: 2 days after treatment).

The HPPD gene copy number and constitutive and inducible expression levels relative to the ALS and CPS genes are shown in Fig 3 and Table 1. Low levels of variation in relative gene copy numbers were observed between plants within the SEN and NEB populations. The ratios in HPPD copy numbers for NEB vs SEN relative to the CPS and ALS genes were 1.04 and 1.12, respectively. Corresponding *p*-values were above the threshold for significance at the 5% significance level, indicating that HPPD gene duplication is not linked to resistance to mesotrione in NEB. In contrast to gene copy numbers, the HPPD gene expression levels varied appreciably from plant to plant within the SEN and NEB populations (Fig 3). This was true when expressed relative to both the CPS and ALS reference genes and for RNA samples extracted from leaf tissues before and after mesotrione treatment. Importantly, comparable *HPPD* expression levels were computed for NEB and SEN both before and after mesotrione treatment. This indicates that resistance to mesotrione in NEB is not due to higher levels of the target-site gene that is expressed constitutively or inducibly following mesotrione application at 48 HAT.

**Table 1:** Average HPPD gene copy number and expression relative to ALS and CPS genes for the SEN and NEB populations.

Sample	Gene comparison	SEN	NEB	Ratio (NEB vs SEN)	P-value
DNA	HPPD vs CPS	1.07	1.11	1.04	0.5165
DNA	HPPD vs ALS	0.92	1.03	1.12	0.0582
RNA before treatment	HPPD vs CPS	0.36	0.63	1.75	0.1379
RNA before treatment	HPPD vs ALS	0.63	1.04	1.73	0.0615
RNA 48 H after treatment	HPPD vs CPS	1.49	1.52	1.02	0.9477
RNA 48 H after treatment	HPPD vs ALS	0.98	1.04	1.06	0.8413

#### ^14^C uptake and translocation

**Fig 4:**
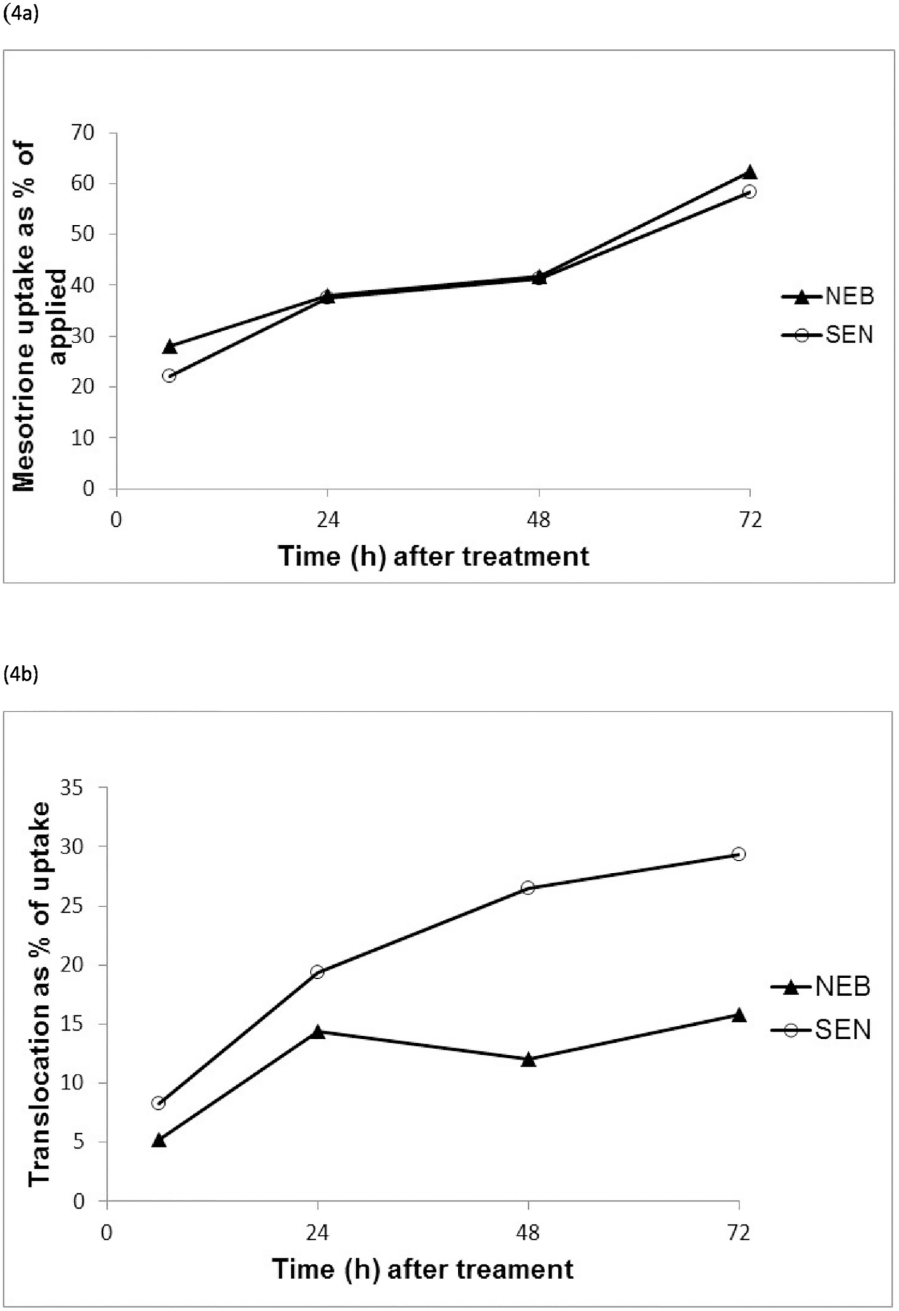
Relative mesotrione (a) uptake and (b) translocation outside the treated area in the NEB and SEN populations.

On average, around 25% of mesotrione applied was absorbed six hours after treatment. The level of mesotrione uptake increased steadily to reach 60% at the end of the time course experiment (Fig 4A). There was no evidence of a difference in mesotrione uptake between NEB and SEN based on the factorial analysis across time (p-value = 0.609). To determine whether differential translocation could account for resistance in NEB, the samples were sectioned into treated leaf, meristem, rest of foliage and root and stem. Radioactivity was predominantly recovered in the treated leaf for both the SEN and NEB populations throughout the experiment (Table 2). Nonetheless, relatively more radioactivity was retrieved outside the treated area for the SEN compared to the NEB population (*p* = 0.014) (Fig 4B). The significant difference in radioactivity recovery was mostly accounted for by the different amounts detected in the meristematic tissues (*p* = 0.0003). For instance, at 48 hours after treatment, 15.4% radioactivity was detected in the meristem of the SEN population and only 4.4% in NEB. Significantly higher levels of radioactivity were also observed in the rest of the foliage for SEN in comparison to NEB (*p* = 0.014) although the difference was not as pronounced as for the plant meristems.

**Table 2:** Means and 95% confidence limits for radiochemical as % of absorbed in the standard sensitive (SEN) and mesotrione resistant population (NEB).

Time after treatment	6h		24h		48h		72h	
Population	SEN	NEB	SEN	NEB	SEN	NEB	SEN	NEB
Treated leaf	91.7 (83.0–97.5)	94.8 (87.4–99.1)	80.7 (69.2–90)	85.6 (75.2–93.6)	73.5 (61.1–84.3)	88.0 (78.1–95.2)	70.7 (58.0–81.9)	84.1 (73.3–92.6)
Meristem	2.6 (0.6–6.2)	1.2 (0.1–3.9)	11.5 (6.5–17.7)	6.1 (2.5–11.0)	15.4 (9.6–22.3)	4.4 (1.5–8.8)	13.8 (8.3–20.5)	5.2 (2.0–9.8)
Stem & root	2.2 (0.4–5.5)	2.2 (0.4–5.4)	2.0 (0.3–5.2)	2.8 (0.6–6.4)	1.5 (0.1–4.4)	2.3 (0.4–5.7)	1.7 (0.2–4.8)	1.4 (0.1–4.2)
Rest of foliage	3.3 (0.8–7.5)	1.7 (0.1–5.0)	5.5 (2.0–10.5)	5.4 (2.0–10.3)	9.2 (4.6–15.3)	5.2 (1.9–10.1)	13.2 (7.5–20.1)	8.7 (4.2–14.7)

#### Unlabelled mesotrione metabolism

**Fig 5:**
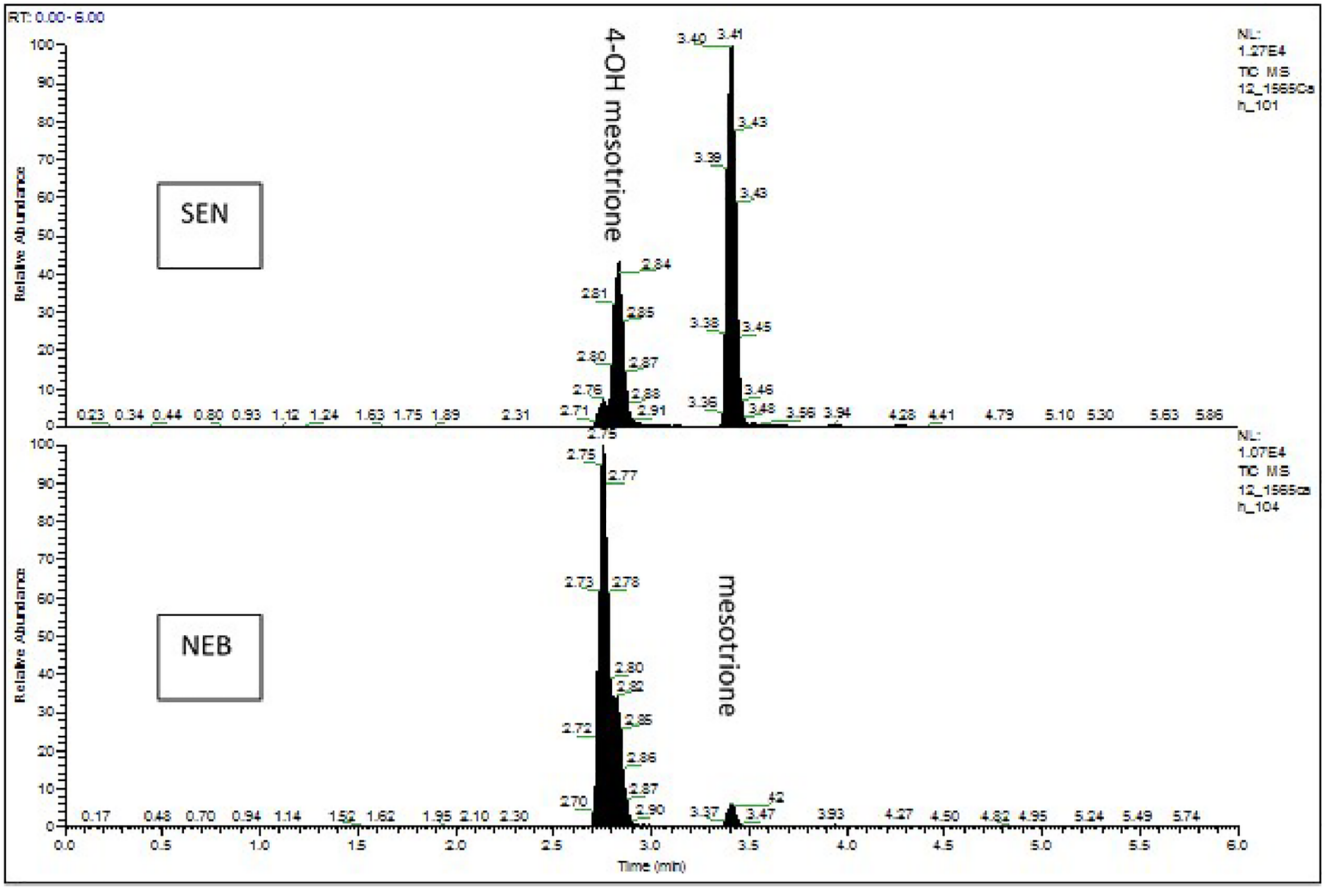
Typical HPLC chromatogram showing mesotrione and its major metabolite 4-hydroxymesotrione in the SEN and NEB populations (72 hours after treatment).

**Fig 6:**
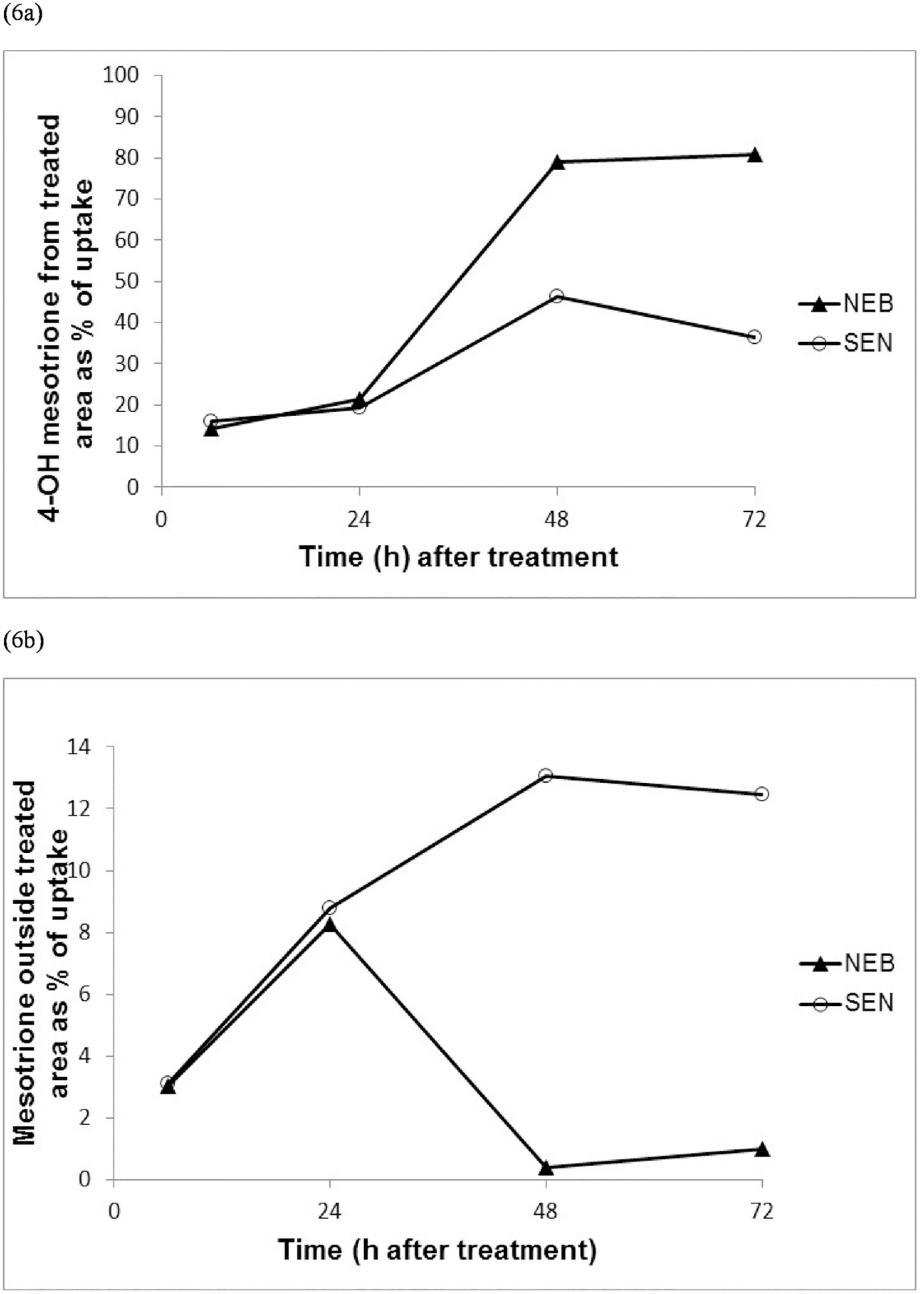
Relative (a) 4-OH mesotrione metabolite in treated leaf and (b) parent mesotrione outside treated leaf in the NEB and SEN populations.

Under our HPLC conditions, mesotrione and its polar metabolites, 4-hydroxymesotrione and AMBA, could be clearly resolved with retention times of 3.5 min, 2.9 min and 2.3 min respectively. Typical HPLC profiles for one each of NEB and SEN plants are provided in Fig 5. The AMBA peak is not visible on the profile given the low levels of this compound detected among all the plant samples analysed. Factorial analysis of the levels of mesotrione and 4-hydroxymesotrione shows clear evidence that the differences between NEB and SEN are time-dependent (p << 0.05) (Table 3 and Fig 6A). Individual t-tests carried out at the separate time points show no convincing evidence of any population differences at the 6h and 24h assessment times (P >> 0.05) but clear evidence of differences at the 48h and 72h assessments (P << 0.05). In this respect, the amount of parent mesotrione remaining in the treated area was as high as 38.1% of total applied for SEN and only 7.5% for NEB at 72 hours. Consequently, relatively higher levels of 4-hydroxymesotrione were detected in the NEB (80.8%) vs the SEN (36.3%) population in the treated leaves. A similar scenario unfolded when mesotrione and its metabolites were quantified in the rest of the plants with significantly higher levels of the parent compound detected for SEN in comparison with NEB at the 48h and 72h but not earlier time points (Table 3 and Fig 6B). For example, 12.5% of applied mesotrione was recovered in the rest of the plant for the SEN population and only 1% for NEB at the 72 hour assessment time.

**Table 3:** Means and 95% confidence limits for mesotrione and metabolites as % of uptake as measured by LCMS.

Time after treatment	6h		24h		48h		72h	
Population	SEN	NEB	SEN	NEB	SEN	NEB	SEN	NEB
Mesotrione—treated area	78.1 (68.8–86.1)	79.7 (70.7–87.5)	67.3 (57.1–76.7)	65.7 (55.4–75.2)	26.8 (18.0–36.5)	11.8 (5.9–19.4)	38.1 (28.2–48.5)	7.5 (3.0–14.0)
Mesotrione—rest of plant	3.1 (1.2–5.9)	3.0 (1.2–5.7)	8.8 (5.4–12.9)	8.3 (5.0–12.3)	13.0 (8.9–17.8)	0.4 (0.0–1.7)	12.5 (8.4–17.2)	1.0 (0.1–2.7)
4-hydroxy—treated area	16.2 (9.1–24.8)	14.4 (7.7–22.7)	19.2 (11.5–28.4)	21.3 (13.2–30.8)	46.3 (35.7–57.0)	78.9 (69.6–87)	36.3 (26.3–46.9)	80.8 (71.7–88.5)
4-hydroxy—rest of plant	0.4 (0.0–1.1)	0.5 (0.1–1.3)	1.4 (0.5–2.6)	1.3 (0.5–2.6)	8.0 (5.7–10.6)	3.3 (1.9–5.1)	9.2 (6.8–12.0)	4.4 (2.7–6.4)
AMBA—treated area	1.8 (0.9–3.0)	1.9 (1.0–3.2)	3.1 (1.8–4.6)	2.9 (1.7–4.3)	5.1 (3.5–7.0)	4.6 (3.1–6.5)	2.6 (1.5–4.1)	4.0 (2.6–5.7)

## Discussion

### Evolution of resistance to post-emergence application of mesotrione

Field resistance to mesotrione applied post-emergence was confirmed in an *A*. *tuberculatus* population from Nebraska via whole plant pot assays conducted under controlled glasshouse conditions. The estimated RI (45.5) was relatively high compared to those determined for two other *A*. *tuberculatus* populations from Illinois (10–35 fold resistance increase depending on the sensitive population used) and Iowa (RI = 8) [26, 28]. The difference in the resistance indices in the three populations could be due to diverse sets of resistance genes and expression levels involved or different proportions of sensitive and recalcitrant seeds collected from field survivors.

Analysis of field treatment histories reveals that the Nebraska and Iowa sites were in seed corn/soybean rotation in alternate years whilst the Illinois population was in continuous seed corn production for seven years prior to resistance being confirmed in the different *A*. *tuberculatus* samples [26, 28]. In any case, resistance in the three populations evolved relatively quickly, very likely because HPPD herbicides were highly relied upon for controlling emerged and possibly large (> 10 cm) *A*. *tuberculatus* plants in the seed corn production years. Inbred seed corn plants are generally not as competitive and are often more damaged by herbicide applications compared to hybrid field corn varieties [33]. Consequently, relatively large *Amaranthus* spp. plants are allowed to proliferate and accumulate ‘creeping’ resistance genes which, on their own, would not be sufficient to permit the individuals to survive an HPPD herbicide treatment but when accumulated in a few subsequent generations would lead to resistance to HPPD and other herbicides [34]. The multi-genic and complex nature of resistance to mesotrione is suggested from classical genetics studies in the HPPD recalcitrant *A*. *tuberculatus* population from Illinois [35] whilst the mode of inheritance and potential number of resistant genes remain to be determined in the Iowa and Nebraska populations.

Overall, resistance to HPPD herbicides in Midwestern USA has evolved slower and is not as problematic as with other single-site herbicide modes of action such as ALS, photosystem II and EPSPS inhibitors [25]. After more than 15 years of intensive HPPD herbicide use, resistance is fully established in only five *A*. *tuberculatus* and three *A*. *palmeri* populations since the first reported case in McLean County, Illinois, in 2009 [26]. More recently, a random survey of 187 samples from Missouri has identified three additional *A*. *tuberculatus* populations that survived a single discriminative rate of mesotrione in a glasshouse experiment [36]. Additional dose response tests using larger number of individuals per herbicide rate are required to confirm the resistance status of these latter three populations. The few instances of resistance to HPPD-inhibiting herbicides in corn agro-systems in the USA may be explained by the fact that they are typically used in two, three and even four-way mixtures with compounds belonging to other modes of action. One of the preferred mixing partner is the PSII-inhibitor, atrazine, which acts synergistically with HPPD herbicides [22]. Synergism between these two herbicide modes of action has been demonstrated in PSII-sensitive, PSII-resistant and PSII/HPPD resistant populations, thereby contributing to the long-term sustainability of HPPD herbicides for controlling *Amaranthus* spp. [23, 24, 27]. Of concern, however, is the fact that the few *A*. *tuberculatus* and *A*. *palmeri* populations that are resistant to HPPD herbicides are also recalcitrant to two, three and even four other herbicide modes of action, consistent with the ability of these two dioecious and highly prolific species to accrue resistance to different classes of herbicides [25, 37, 38]. All the populations that were not controlled with HPPD compounds were also resistant to PSII and ALS herbicides, as these products have been widely used in corn/soybean production in Midwestern USA for over 25 years. In addition to HPPD, PSII and ALS inhibitors, the latest *A*. *tuberculatus* population identified in Champaign County, Illinois, is also recalcitrant to protoporphyrinogen oxidase inhibitors as well as synthetic auxin herbicides [39]. This scenario seriously limits the number of effective chemical options for managing such multiple resistant weed populations.

### Mechanism of resistance to mesotrione applied post-emergence

Detailed mechanism studies have showed that resistance to mesotrione in the Nebraska population is not due to a target-site mutation or HPPD gene duplication, in agreement with previously published data in other *A*. *tuberculatus* and *A*. *palmeri* populations [30, 31]. The absence of a target-site resistance mutation in all the *Amaranthus* spp. populations investigated to date contrasts with what was observed with other single-site herbicide modes of action following similar use intensity. Target-site insensitivity due to a subtle amino acid change in *Amaranthus* spp. was documented for ALS, PSII, PPO and EPSPS inhibitors only after a few years of widespread usage [40–44]. The lack of evolved HPPD target-site resistance mutations may be explained by the fact that HPPD herbicides are competitive inhibitors and the target enzyme may not tolerate many amino acid changes without compromising catalytic activity [16, 45]. Additionally, mutagenesis studies in view of engineering HPPD tolerance in dicotyledonous crops have shown that individual mutations are not sufficient to confer resistance to HPPD herbicides as is the case for some ALS and PSII resistance mutations [46, 47]. Indeed, target gene mutations and over-expression had to be introduced in an already tolerant monocotyledonous *HPPD* to endow sufficient levels of resistance to HPPD herbicides in soybean [10, 48, 49]. Another important contributing factor is that HPPD inhibitors are almost always used in mixtures with other overlapping herbicide modes of action, thereby limiting the risk of selecting for an HPPD target-site resistance mutation in *Amaranthus* spp.

Resistance in the Nebraska populations was neither due to constitutive nor mesotrione-inducible over-expression of the HPPD gene. This differs with data generated on two *A*. *palmeri* populations from Kansas and Nebraska whereby mesotrione resistance appeared to be associated, in part, with higher numbers of HPPD transcripts compared to three sensitive populations [31]. The observed increase in target gene expression levels varied from 5–12 depending on the sensitive population being considered [31]. Contrary to gene copy number, the level of HPPD expression as measured by RT-qPCR was quite variable between plants within the SEN and NEB populations. For instance, up to 10-fold difference in HPPD gene expression was detected between resistant NEB plants whilst this figure was as high as 30-fold between two extreme SEN individuals. Unless a sufficiently large number of biological replicates are evaluated, HPPD gene expression data should be treated with caution when drawing conclusions about the potential contribution to resistance to HPPD inhibiting herbicides.

Resistance to mesotrione in NEB is due to enhanced detoxification of the parent compound into 4-hydroxymesotrione, thus mirroring the selectivity basis of the HPPD herbicide in naturally tolerant corn [2, 16]. The cytochrome p-450 mediated hydroxylation occurs so rapidly in the crop that translocation outside the treated area is limited. In contrast, slow metabolism in sensitive weeds allows ample translocation of mesotrione to other parts of the plant by both acropetal and basipetal movement. Cytochrome p-450 mediated metabolism via hydroxylation of dione ring at the 4’ position was also found to account for resistance to mesotrione in the *A*. *tuberculatus* population from Mclean County, Illinois [30]. Convergent resistance by the same mechanism in these two distant populations attests for the growing evidence that resistance in highly heterogeneous and prolific weed species such as *A*. *tuberculatus* and *A*. *palmeri* occurs primarily by spontaneous evolution from standing genetic variation in the field rather than by migration from an initial location [50]. Whilst increased detoxification of mesotrione at the treated area was clearly established in NEB, it remains to be determined whether resistance could also be due to impaired translocation of the parent compound to other plant parts as well. Indeed, lower levels of radioactivity were detected outside the treated area and in particular in the meristematic tissues for NEB compared to SEN. The difference in radioactivity levels outside the treated area could be due to reduced mesotrione translocation, as is unambiguously demonstrated for other herbicides such as glyphosate and paraquat in various resistant grass or broadleaf weeds and more recently as documented for 2,4-D in wild radish [51–54]. Conclusion of impaired transport in the latter studies was facilitated by the fact that differential metabolism of glyphosate, paraquat and 2,4-D was not a contributing factor in resistance. Resolution of the potential contribution of reduced translocation of mesotrione in NEB could be achieved by analysing the fate of other experimental HPPD inhibitors sharing similar physico-chemical properties to mesotrione but blocked and metabolically robust on the aryl-dione ring [55].

### Cross or multiple resistance to foliar-applied HPPD herbicides

The Nebraska *A*. *tuberculatus* population was multiply resistant to tembotrione and topramezone applied post-emergence. The cross-resistance between foliar-applied HPPD inhibiting herbicides is in line with what was observed for some other *A*. *tuberculatus* and *A*. *palmeri* populations [26, 27]. When applied post-emergence at their commercial field rates, mesotrione, tembotrione and topramezone provided unsatisfactory control (27%, 31% and 58% respectively) for the HPPD resistant *A*. *tuberculatus* population from Illinois (MCR) whilst the two reference populations used for comparison were completely killed [26]. Similarly, a 4-23- fold resistance increase was estimated for mesotrione, tembotrione and topramezone for the *A*. *palmeri* population from Nebraska [27]. Since both mesotrione and tembotrione were used for dicotyledonous weed control in the Nebraska field, they may have co-selected for resistance to HPPD herbicides in NEB. Tembotrione and mesotrione belong to the same triketone HPPD herbicide subgroup and as such share a similar chemical structure and liability with regard to metabolism, especially on the aryl-dione moiety [2, 11]. We therefore hypothesize that resistance to tembotrione in NEB also occurs by increased detoxification by 4-hydroxylation on the dione ring, very similar to what was established for mesotrione. This idea is supported by a genetic study on a cross between two corn varieties that are sensitive and resistant to HPPD herbicides [56]. Analysis of the progeny identified a single major resistance locus and, importantly, a close linkage between tolerance to mesotrione and tembotrione in corn.

It is noteworthy that NEB was never pressured with topramezone in the field, yet significant levels of resistance were observed for this HPPD inhibitor as well. It therefore appears that the gene(s) selected by mesotrione and/or tembotrione has conferred resistance to topramezone in the Nebraska population. Topramezone belongs to the pyrazolone HPPD herbicide sub-class and the selectivity basis in corn is primarily through *N*-demethylation at the pyrazole ring [14]. It remains to be determined whether NEB impersonates corn and degrades topramezone by *N*-demethylation or via ring or alkyl hydroxylation at a liable position, as is the case for mesotrione.

### Control of NEB with HPPD herbicides applied pre-emergence

A modest resistance index of 2.4 was computed between the Nebraska and the standard sensitive population when mesotrione was applied pre-emergence. NEB was fully controlled at half the recommended field rate of soil-applied mesotrione whilst plant survivors were recorded at up to 4X the commonly use rate of the herbicide applied post-emergence. Similarly, NEB was killed within the range of recommended rates of isoxaflutole applied pre-emergence. Therefore, it appears that the metabolic resistance mechanism to HPPD herbicides identified in emerged *A*. *tuberculatus* plants is not significantly expressed at the seed germination stage. An improvement in weed control was also observed when mesotrione was applied post-emergence on smaller and more vulnerable individuals compared to plants at later growth stages for the *A*. *tuberculatus* from Illinois [57]. The same was true for several other herbicides that are effective on *Amaranthus* spp. For instance, atrazine was more effective on a GST-based metabolic resistant *A*. *tuberculatus* population from Illinois when applied pre-emergence as opposed to post-emergence [58]. Up to a 10-fold gain in PPO herbicide efficacy was also reported for a target-site (210 codon deletion) resistant *A*. *tuberculatus* population treated at pre-emergence as opposed to 7 cm tall plants [59]. Therefore, targeting populations early in the season when the plants are small, or even more so at the seed germination stage, appears to be a good strategy for maintaining the efficacy of HPPD and other herbicides that are active on *Amaranthus* spp..

## Conclusion and future research

We have confirmed high levels of resistance to mesotrione, tembotrione and topramezone applied post-emergence in an *A*. *tuberculatus* population from Nebraska, USA. Mesotrione and isoxaflutole applied pre-emergence are still effective on the NEB population, suggesting that the gene(s) endowing resistance to HPPD herbicides in emerged *A*. *tuberculatus* plants is not appreciably expressed at the seed germination stage in NEB. Resistance due to enhanced metabolic breakdown of mesotrione to 4-hydroxymesotrione has been clearly established, similar to what is documented in corn and the MCR population [30]. It remains to be determined whether other non-target-site resistance mechanisms apply, in particular reduced cellular transport or whole-plant translocation, by exploring the relative movement of metabolically blocked experimental triketones. Given the dissimilar structures and corn selectivity basis between mesotrione/tembotrione and topramezone, further research will investigate the physiological mechanism by which NEB is resistant to the pyrazolone herbicide topramezone.

## Supporting information

S1 File Supporting information file for whole plant dose response, qPCR, uptake, translocation and metabolism tests. (XLSX)
